# Cubic Liquid Crystalline Nanostructures Involving Catalase and Curcumin: BioSAXS Study and Catalase Peroxidatic Function after Cubosomal Nanoparticle Treatment of Differentiated SH-SY5Y Cells

**DOI:** 10.3390/molecules24173058

**Published:** 2019-08-22

**Authors:** Miora Rakotoarisoa, Borislav Angelov, Shirly Espinoza, Krishna Khakurel, Thomas Bizien, Angelina Angelova

**Affiliations:** 1 Institut Galien Paris-Sud, CNRS UMR 8612, Univ. Paris-Sud, Université Paris-Saclay, LabEx LERMIT, F-92290 Châtenay-Malabry CEDEX, France; 2 Institute of Physics, ELI Beamlines, Academy of Sciences of the Czech Republic, Na Slovance 2, CZ-18221 Prague, Czech Republic; 3 Synchrotron SOLEIL, l’Orme des Merisiers, Saint-Aubin - BP 48, 91192 Gif-sur-Yvette CEDEX, France

**Keywords:** liquid crystalline nanoparticles, cubosome, catalase, curcumin, fish oil, BioSAXS, peroxidatic activity of catalase

## Abstract

The development of nanomedicines for the treatment of neurodegenerative disorders demands innovative nanoarchitectures for combined loading of multiple neuroprotective compounds. We report dual-drug loaded monoolein-based liquid crystalline architectures designed for the encapsulation of a therapeutic protein and a small molecule antioxidant. Catalase (CAT) is chosen as a metalloprotein, which provides enzymatic defense against oxidative stress caused by reactive oxygen species (ROS) such as hydrogen peroxide (H_2_O_2_). Curcumin (CU), solubilized in fish oil, is co-encapsulated as a chosen drug with multiple therapeutic activities, which may favor neuro-regeneration. The prepared self-assembled biomolecular nanoarchitectures are characterized by biological synchrotron small-angle X-ray scattering (BioSAXS) at multiple compositions of the lipid/co-lipid/water phase diagram. Constant fractions of curcumin (an antioxidant) and a PEGylated agent (TPEG_1000_) are included with regard to the lipid fraction. Stable cubosome architectures are obtained for several ratios of the lipid ingredients monoolein (MO) and fish oil (FO). The impact of catalase on the structural organization of the cubosome nanocarriers is revealed by the variations of the cubic lattice parameters deduced by BioSAXS. The outcome of the cellular uptake of the dual drug-loaded nanocarriers is assessed by performing a bioassay of catalase peroxidatic activity in lysates of nanoparticle-treated differentiated SH-SY5Y human cells. The obtained results reveal the neuroprotective potential of the in vitro studied cubosomes in terms of enhanced peroxidatic activity of the catalase enzyme, which enables the inhibition of H_2_O_2_ accumulation in degenerating neuronal cells.

## 1. Introduction

**Figure 1:**
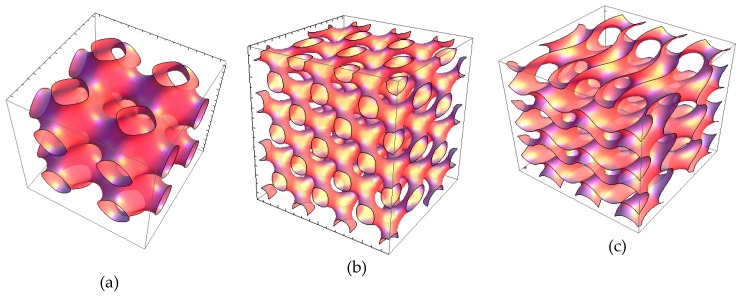
Three-dimensional organizations of cubic liquid crystalline phases: (**a**) Primitive cubic (also referred to as *Im3m*/Q^IIP^), (**b**) bicontinuous double diamond cubic (*Pn3m*/Q^IID^), and (**c**) bicontinuous gyroid cubic (*Ia3d/*Q^IIG^) types.

Self-assembled lipid cubic phase (LCP) architectures comprise bilayer lipid membranes with a three-dimensional (3D) crystalline packing order and periodic networks of aqueous channels (Figure 1) [1,2,3,4,5,6]. The amphiphilic nature of the lyotropic liquid crystalline phases and nanoparticles (LCNPs) makes them suitable for the embedding of either lipophilic or hydrophilic guest compounds [7,8,9,10,11,12,13,14,15,16,17,18,19]. It has been estimated that LCPs have a large surface area of lipid/water interfaces, which is of the order of 400 m^2^/g [7]. Compared to liposome carriers, lipid-based cubosomes, hexosomes and spongosomes involve multiple internal compartments, which represent a structural advantage enabling an enhanced encapsulation efficacy [9,10,11,14,15,16,17,18,19]. The entrapment of biomolecules of various dimensions and hydrophilicities is achievable in such nanocarriers as well as their sustained release [10,12]. For example, LCPs have been used to encapsulate proteins of different concentrations and sizes from Cyt C (12 kDa) to fibrinogen (340 kDa) [8,11,20,21,22,23,24,25,26,27]. High encapsulation efficacy has been reported for hydrophilic guest macromolecules such as brain-derived neurotrophic factor (BDNF), ovalbulmin and protein vaccines [11,20,21,22,23,24]. Soluble, peripheral, and integral membrane proteins have been studied in LCPs in relation to in meso protein crystallization, biosensor development involving encapsulated enzymes, and drug-delivery systems [21,22,23,24,25,26,27,28]. LCP-derived nanocarriers are increasingly used in a range of applications employing lipophilic drugs and theranostic agents [14,15,16,17,18,19]. The bioavailability of anti-inflammatory (flurbiprofen) [14], antiretroviral (efavirenz) [15], and anticancer (paclitaxel) [16] agents, as well as of antioxidants such as curcumin [17,19], has been considerably improved thanks to their protection and transport by the host cubic liquid crystalline phases [18].

Regarding the lipid polymorphism, liquid crystalline phases have been formed by the self-assembly of hydrated mixtures of lyotropic lipids, co-lipids (oils or surfactants), and an aqueous phase, which may contain dissolved biomolecules (e.g., proteins, peptides or nucleic acids) [1,2,3,4,5,6,7,18,19,20,21,22,23,24,25,26,27,29,30,31,32,33,34,35,36]. Hydrated non-lamellar lipids (such as monoolein (MO)) can self-assemble into inverted bicontinuous cubic phases, bicontinuous sponge or inverted hexagonal phases depending on the experimental conditions and the applied stimuli [4,5,18,25,29,30,31,32]. Different types of bicontinuous cubic phases have been observed in lipid membranous systems [3,4,5,6,7,25,29]. Primitive (also referred to as *Im3m*/Q^IIP^), double diamond (*Pn3m*/Q^IID^), and gyroid (*Ia3d*/Q^IIG^) cubic phases (Figure 1) are the most common ones for lyotropic monoglycerides. Cubosome nanoparticles are fabricated upon dispersion of the bulk cubic liquid crystalline phases in excess aqueous medium [8,9,13,16,17,18,19,20,24]. The cubosome structure is sensitive to the incorporation of additives such as therapeutic molecules and diagnostic probes required for monitoring of the biomedical response to active targeting [9,16,18,21,35].

It is believed that cubosomal nanostructures co-loaded with a therapeutic protein and a natural antioxidant are safe and provide neuroprotection against oxidative stress and neuronal damage [37]. Currently, nanoscale materials are attracting increasing interest for therapeutic applications in the field of neurological disorders [37,38,39,40,41]. In our recent study, we showed that cubosome lipid nanoparticles loaded with curcumin (CU) and fish oil (FO) have neuroprotective potential against the accumulation of reactive oxygen species (ROS) and H_2_O_2_-induced cell death [19]. Here, we combine curcumin and a therapeutic antioxidant enzyme in such advanced nanostructured lipid carriers.

Catalase (a tetrameric protein formed by 60-kDa monomer units) is a metalloenzyme that catalyzes the dismutation of H_2_O_2_ (a harmful oxidizing agent) to oxygen and water [42,43,44,45,46]. Catalase (CAT) is expressed in all major bodily organs (especially in the liver and kidneys) and in erythrocytes, where it plays an essential role in cell defense against oxidative stress [47,48,49,50]. Polymorphism of the catalase gene is associated with a number of diseases such as diabetes, Alzheimer’s disease, cancer, hypertension, vitiligo, and acatalasemia [51,52,53,54]. It should be emphasized that catalase undergoes rapid elimination from the bloodstream and is characterized by poor intracellular delivery. Thus, catalase exhibits a short half-life and poor operational stability and reusability as an enzyme, which limits its potential therapeutic applications [55,56,57,58,59,60]. The unstable biomacromolecule can be immobilized in order to increase its stability and improve its enzymatic performance, solubility and specificity [61,62,63,64,65,66,67]. Catalase has been immobilized on solid supports from natural polymers (chitosan, gelatin, etc.), synthetic polymers (styrene, methyl methacrylate, acrylamide, etc.), and inorganic particles (calcium carbonate, silica, gold, etc.) [61,62,63,64,65,66,67]. This helped to overcome the enzyme’s degradation or deactivation. Liposomes have been used for intravenous catalase delivery [63].

The present work focuses on the design and characterization of advanced self-assembled liquid crystalline nanostructures of catalase and curcumin in view of a prospective combination therapy for neurodegenerative disorders. Self-assembly properties are investigated by biological synchrotron small-angle X-ray scattering (BioSAXS), which is a high-throughput technique providing valuable structural data from weakly scattering biological solutions in real time [6,35,68,69]. BioSAXS can detect and determine the internal nanostructure, the shape and the structural evolution of various kinds of protein, peptide and lipid-protein assemblies [11,22,32,68,69,70]. We study mixed lipid (MO)/protein (catalase) self-assembly into nanostructures in the presence of a small molecule antioxidant (curcumin). Our first aim is to determine via BioSAXS the structural effect of the catalase enzyme’s inclusion into cubic liquid crystalline assemblies, i.e., catalase-associated cubosomal nanostructures. The second aim of the work is to evaluate the catalase activity after the treatment of neuronally derived SH-SY5Y cells with catalase-associated cubosome nanoparticles.

## 2. Results

### 2.1. Structural Investigation of Liquid Crystalline Assemblies by Synchrotron BioSAXS

#### 2.1.1. Design and Production of Self-Assembled Nanocarriers for the Loading of Catalase and Curcumin

**Figure 2:**
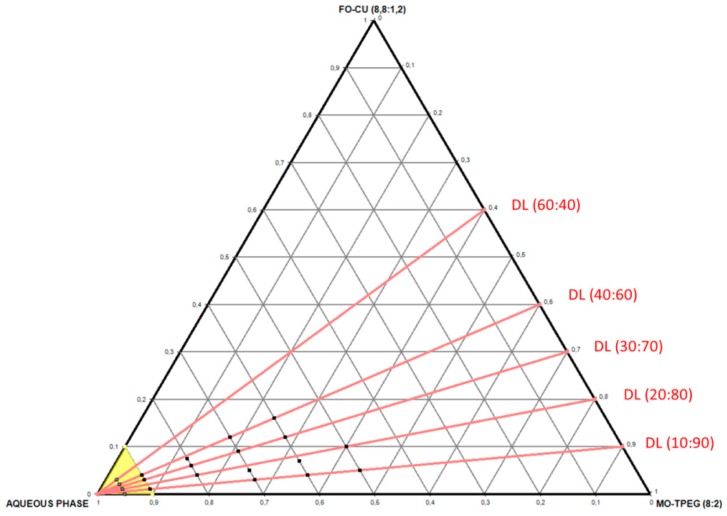
Phase diagram showing the dilution lines for the preparation of nanocarriers composed of monoolein (MO), TPEG_1000_ amphiphile, fish oil (FO), curcumin (CU), and water. The MO/TPEG_1000_ and FO/MO weight ratios are constant (80:20 and 88:12, respectively). The water-rich region is represented by a yellow triangle. The aqueous phase contains catalase (CAT) in the CAT-loaded formulations. The black points indicate the compositions for which experimental biological synchrotron small-angle X-ray scattering (BioSAXS) data are shown in Section 2.1.3 in the sequence from more concentrated to more diluted assemblies: samples C195, C196, C197 and C199 on the dilution line DL 10:90; samples C285, C286, C287 and C288 on the dilution line DL 20:80; samples C376, C377, C378 and C379 on the dilution line DL 30:70; and samples C466, C467, C468 and C469 on the dilution line DL 40:60.

The compositions of the investigated self-assembled MO/TPEG_1000_/FO/CU/CAT systems are chosen from the phase diagram presented in Figure 2. Five dilution lines (DL), denoted as DL 10:90, DL 20:80, DL 30:70, DL 40:60, and DL 60:40 (wt%/wt%), are defined in order to characterize the lyotropic behavior of the multicomponent amphiphilic mixtures of therapeutic significance. The water-rich region corresponds to dispersions of nanocarriers in excess aqueous medium. The lipid-rich region corresponds to bulk liquid crystalline assemblies (Figure 2).

#### 2.1.2. Liquid Crystalline Nanostructure Identification in MO/TPEG1000/FO/CU/CAT Systems by BioSAXS

**Figure 3:**
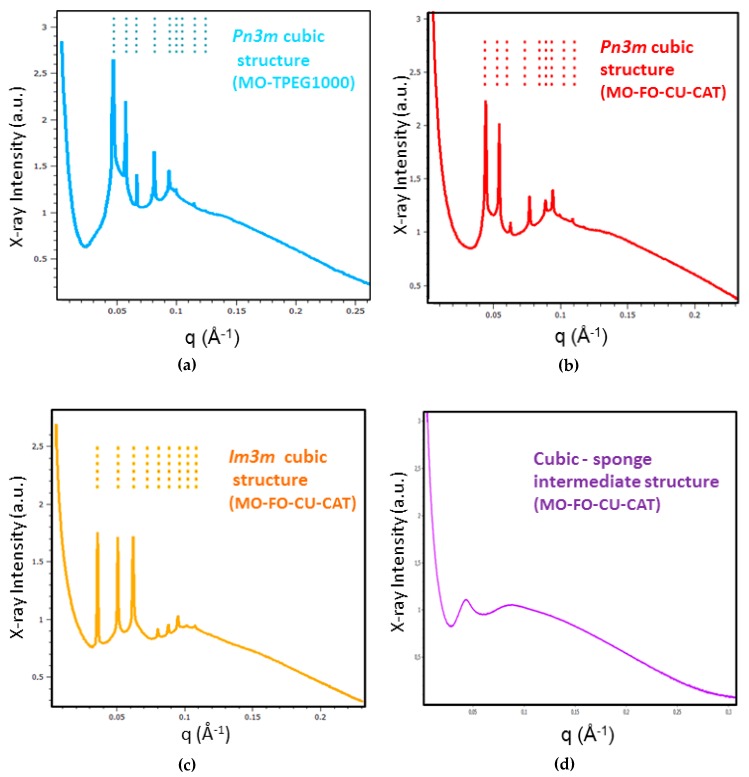
Representative BioSAXS patterns of MO/TPEG_1000_ and MO/TPEG_1000_/FO/CU self-assembled mixtures hydrated in a phosphate buffer or in catalase (CAT) solutions at 22 °C. The recorded Bragg peaks are indicative of the formation of periodic liquid crystalline phases (**a**–**c**): (**a**) MO/TPEG_1000_ lipid mixture hydrated in 0.01 M of phosphate buffer (pH 7.0): the sequence of dashed lines indexes from left to right the (110), (111), (200), (211), (220), (221), (310), (222) and (321) reflections of a diamond cubic lattice (*Pn3m* space group); (**b**) MO/TPEG_1000_/FO/CU/CAT mixture: the dashed lines consecutively denote the (110), (111), (200), (211), (220), (221), (310), (222) and (321) reflections of a *Pn3m* cubic structure, which is preserved in the presence of encapsulated curcumin and catalase protein molecules; (**c**) Pattern of a MO/TPEG_1000_/FO/CU/CAT mixture with indexed (110), (200), (211), (220), (310), (222), (321), (400) and (330) reflections of a primitive cubic lattice (*Im3m* space group); and (**d**) Pattern lacking well-defined Bragg reflections and corresponding to a cubic intermediate formed by the MO/TPEG_1000_/FO/CU/CAT mixture. The sample list is given in Section 4.2.

BioSAXS patterns were recorded at room temperature (22 °C) for all amphiphilic compositions indicated in the diagram in Figure 2. Representative BioSAXS plots revealing the presence or absence of long-range periodicities in the MO/TPEG_1000_/FO/CU three-dimensional (3D) assemblies are shown in Figure 3. The performed structural investigation established that the blank MO/TPEG_1000_ nanocarriers display a long-range 3D periodicity (Figure 3a). The latter was identified by a set of Bragg peaks with q-vector positions spaced in the ratio √2: √3: √4: √6: √8: √9: √10: √12: √14. These Bragg peaks are assigned to the (110), (111), (200), (211), (220), (221), (310), (222), and (321) reflections of a double diamond cubic lattice Q^IID^ (Pn3m space group). Similarly to pure monoolein, which forms a diamond-type (Pn3m) cubic phase in the excess water phase [25,30,39], the lyotropic MO/TPEG_1000_ liquid crystalline assemblies yielded cubic structures under the investigated hydration conditions.

The self-assembled MO-TPEG_1000_-FO-CU-CAT mixtures (see Section 4.2 and Section 4.3 of Methods) displayed two kinds of mesophase structures with long-range 3D periodicities depending on the amount of the included oil (FO) and water. Bicontinuous double diamond cubic Pn3m (Figure 3b) and primitive cubic Im3m phases (Figure 3c) were identified as well as a cubic intermediate from the structural transition to a sponge phase at 22 °C (Figure 3d). Figure 3c shows the set of Bragg peaks with q-vector positions spaced in the ratio √2: √4: √6: √8: √10: √12: √14: √16: √18. These peaks are assigned to (110), (200), (211), (220), (310), (222), (321), (400) and (330) reflections of a primitive cubic lattice Q^IIP^ (Im3m space group). The increase in the incorporated oil amount in the lipid membrane led to a more weakly ordered cubic structure, which coexisted with sponge-type membranes. The resulting pattern is attributed to an intermediate mesophase state owing to the composition-trigerred structural transition (Figure 3d) [2,39].

#### 2.1.3. Characterization of Liquid Crystalline Bulk Structures by BioSAXS

**Figure 4:**
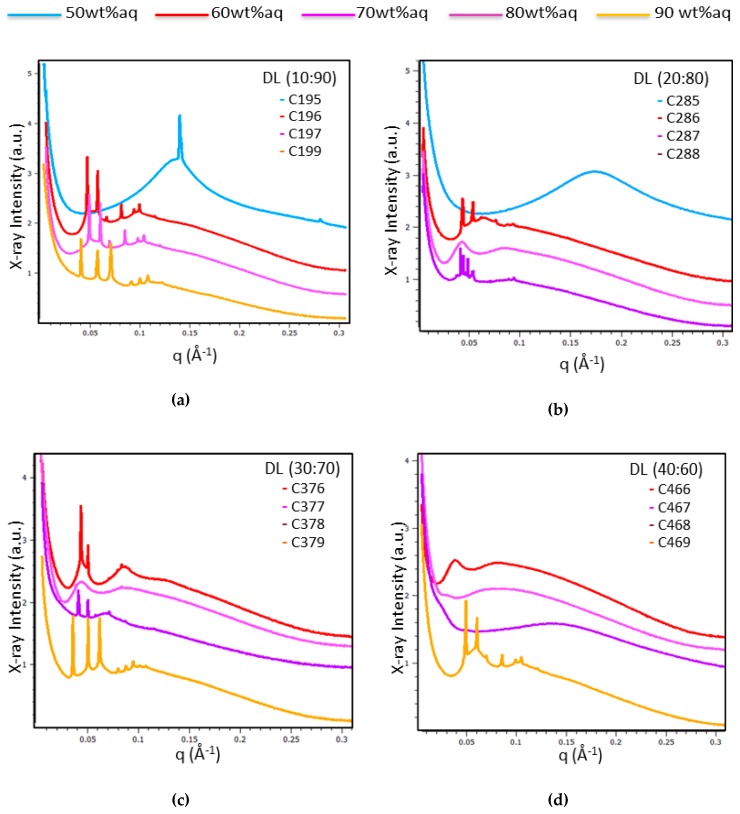
BioSAXS patterns of MO/TPEG_1000_/FO/CU/CAT assemblies acquired at 22 °C along the dilution lines (DL): (**a**) DL = 10:90; (**b**) DL = 20:80; (**c**) DL = 30:70; and (**d**) DL = 40:60 from Figure 2 (for the precise compositions see the points drawn in Figure 2). The structural phase behavior is examined with samples prepared using a 5-mg/mL catalase solution.

Liquid crystalline MO/TPEG_1000_/FO/CU assemblies were prepared using four different ratios, 10:90, 20:80, 30:70 and 40:60 (wt%/wt%), for the fish oil/monoolein mixtures hydrated by a catalase solution (5 mg/mL) (see the compositions indicated in Figure 2 and Section 4.2. The performed BioSAXS experiments were aimed at determining the lipid ratio corresponding to the highest fish oil content, which conserves the cubic mesophase formation at room temperature (22 °C). Figure 4 presents the BioSAXS patterns characterizing the phase behavior at varying water contents along the dilution lines defined in Figure 2. Three major cases were distinguished: (i) well-ordered lipid/protein assemblies of inner cubic lattice symmetries, (ii) cubic mesophases displaying a coexistence of distinct nanodomains, and (iii) cubic intermediates as precursors of a sponge-membrane phase, which is favored by the increase in the fish oil (FO) content.

Figure 4 shows that large domains of ordered cubic phase organization are formed by the self-assembled mixtures C196, C197, and C199 along the dilution line DL = 10:90 (Figure 4a); C286 and C288 along the dilution line DL = 20:80 (Figure 4b); C376, C378, and C379 along the dilution line DL = 30:70 (Figure 4c), and C469 at DL = 60:40 (Figure 4d). At variance, samples C287 at DL = 20:80 (Figure 4b), C377 at DL = 30:70 (Figure 4c), and C466 at DL = 40:60 (Figure 4d) displayed BioSAXS patterns of weakly ordered cubic phases and the onset of the formation of a sponge membrane phase. The mixtures C467 and C468 (obtained along the dilution line DL = 40:60) formed cubic intermediates of the structural transition to a dominant sponge phase (Figure 4d, pattern C468).

The effect of the variation of the hydration level in the MO/TPEG_1000_/FO/CU/CAT systems (Figure 4) pointed out that the structural phase behavior is dominated by a bicontinuous cubic phase formation at fish oil/monoolein ratios of 10:90, 20:80, and 30:70 (wt%/wt%) for water contents between 60 wt% and 80 wt% (see the compositions in Section 4.2). At 90 wt% water content, stable bicontinuous cubic phases were formed at fish oil/monoolein weight ratios from 10:90 to 30:70, and up to 40:60 (wt%/wt%). The MO/TPEG_1000_/FO/CU/CAT system formed a primitive cubic *Im3m* phase at 90 wt% water content, whereas the bicontinuous cubic *Pn3m* phase was most stable at 60 wt% to 80 wt% water contents.

In addition, the hydration of the lipid mixtures by the CAT protein solution at 50 wt% water content (C195, C285) resulted in the formation of lamellar and sponge phases depending on the FO/MO weight ratio (Figure 4a,b, respectively). An intermediate cubic phase domain preceded the induction of a dominant sponge phase at a fish oil/monoolein ratio of 40:60 wt%/wt%. The determined internal structures and lattice constants of the studied MO/TPEG_1000_/FO/CU/CAT self-assembled architectures are shown in Table 1.

The unit lattice parameters of the cubic structures, a(_Q_), were calculated from the reciprocal slope of the linear plots *q* versus (h^2^ + k^2^ + l^2^)^1/2^, where (*hkl*) are the Miller indices of the recorded Bragg peaks (equation (1)).

*q* = (2π/a_(Q)_) (*h*^2^ + *k*^2^ + *l*^2^)^1/2^ (1)

The results for the structural parameters of the bulk cubic liquid crystalline structures are presented in Table 1.

#### 2.1.4. BioSAXS Characterization of Nanocarrier Dispersions

Aqueous dispersions of liquid crystalline nanocarriers were prepared at fish oil/monoolein weight ratios of 0:100, 20:80, 40:60 and 60:40 (wt%/wt%) and a constant water content (95 wt%). Catalase-free (i.e., blank) and catalase (CAT)-loaded nanocarriers were investigated by BioSAXS at the same compositional proportions of the lipid ingredients (see Section 4.3 of Methods for the amphiphilic compositions). This permitted the evaluation of the structural effect of the catalase association to the lipid nanocarriers by small-angle X-ray scattering.

**Figure 5:**
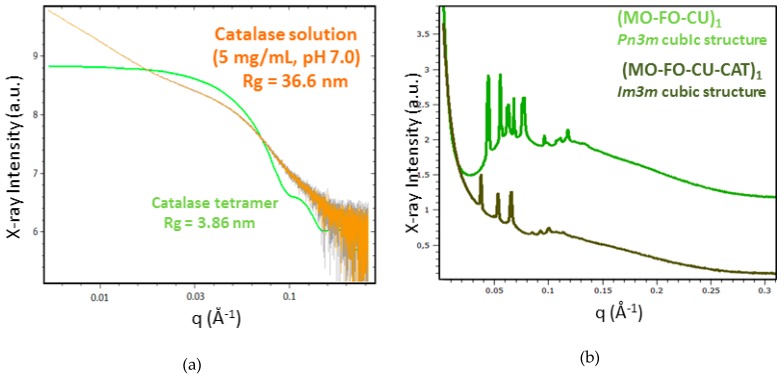

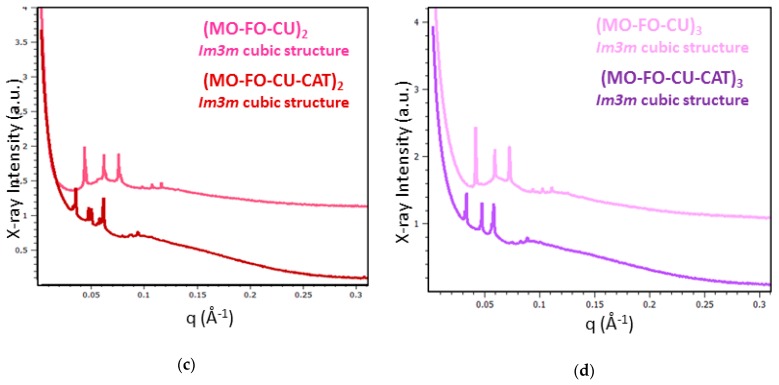
BioSAXS patterns of (**a**) catalase solution (5 mg/mL) in 0.01 M of phosphate buffer, and (**b–d**) liquid crystalline nanoparticle dispersions obtained in the presence or absence of catalase (CAT) at varying fish oil (FO)/monoolein (MO) ratios (wt%/wt%): (**b**) 20:80 (MO-FO-CU)_1_ vs. (MO-FO-CU-CAT)_1_; (**c**) 40:60 (MO-FO-CU)_2_ vs. (MO-FO-CU-CAT)_2_; and (**d**) 60:40 (MO-FO-CU)_3_ vs. (MO-FO-CU-CAT)_3_. The percentages of curcumin (CU) and TPE_G1000_ are constant with regards to MO and FO (see the compositions in Section 4.3 of Methods Temperature is 22 °C.

A synchrotron BioSAXS plot of a catalase solution is shown in Figure 5a for a protein concentration of 5 mg/mL, which was employed for CAT entrapment in nanocarriers. The experimental curve shifts with regard to that determined for non-interacting catalase tetramers (green plot). The estimated radius of gyration (Rg = 36.6 nm) appears to be nearly ten times larger than that of an isolated catalase tetramer (Rg = 3.86 nm). This result suggests that the protein CAT forms oligomeric structures above a certain solution concentration. Several proteins have a tendency to aggregate in aqueous medium. This is an important problem in biotechnology and the pharmaceutical industry. Proteins in an aggregated state generally do not have the same biological activity as proteins in a native state. The immobilization of catalase is of significant interest for the enhancement of its stability and improving its enzymatic performance. We interpreted the BioSAXS data about CAT aggregation as oligomers because the formed aggregates do not have microscopic sizes. The results also suggest that CAT may show a preference for interaction with the nanostructured lipid phase as the latter can provide interfaces of different polarities and less hydrophilic compartments for embedding the protein.

Figure 5b–d shows the BioSAXS patterns of catalase-free and catalase-loaded nanoparticles samples at three selected (MO-FO-CU) ratios (Section 4.3). Well-defined Bragg peaks of cubic liquid crystalline structures are detected at 22 °C both in the absence and in the presence of catalase protein. Figure 5b demonstrates that the initial bicontinuous *Pn3m* cubic organization of the blank nanocarriers (MO-FO-CU)_1_ is transformed into a primitive cubic *Im3m* structure upon addition of CAT at a FO/MO ratio of 20:80 wt%/wt% in the formulation. In the absence of CAT loading, the (MO-FO-CU)_1_ cubosome nanoparticles involved coexisting domains of the *Pn3m* space group with two distinct cubic unit cells dimensions. The *Pn3m* unit lattice parameters estimated for these cubosome particles are a_1_(_Q_) = 16.4 nm and a_2_(_Q_) = 20.0 nm, respectively (Table 2). The CAT-loaded cubosome particles (MO-FO-CU-CAT)_1_ (Figure 5b) were characterized by the *Im3m* space group and displayed a bigger cubic lattice parameter, a(_Q_) = 23.2 nm. At 95 wt% water content, the increase in the FO/MO weight ratio to 40:60 and 60:40 (wt%/wt%) yielded predominantly primitive cubic *Im3m* inner structures (Figure 5c,d).

The cubic unit cell dimensions of the liquid crystalline nanoparticles were estimated from the BioSAXS results using equation (1). Table 2 shows that the cubic lattice parameters in the catalase-free nanocarriers increase with the increase in the fish oil and curcumin amounts. The values range from a(_Q_) = 16.4 nm (*Pn3m* inner cubic structure) to a(_Q_) = 21.3 nm (*Im3m* inner cubic structure). These unit cell magnitudes result from the fragmentation of the drug-loaded lyotropic lipid cubic phase by the PEGylated dispersion agent in excess aqueous medium. They differ from that typical for the pure MO bulk lipid cubic phase (a_(Q)_ = 10.5 nm) [39]. The increased cubic lattice parameter of the catalase-loaded nanoparticles (a_(Q)_ = 26.3 nm) as compared to nanoparticles without catalase (a_(Q)_ = 21.3 nm) can be explained by the entrapment of the protein macromolecules, which may cause swelling of the monoolein liquid crystalline structures [2,8].

The investigated nanodispersions were also characterized by quasi-elastic light scattering (QELS) measurements. The sizes of the particles determined from their volume distributions in the samples are shown in Table 2. The mean hydrodynamic size of the catalase-free nanoparticles increases with the increase in the fish oil and curcumin proportions (see Section 4.3 of Methods). The values vary between 106 nm and 255 nm. The association of catalase to the lipid nanoparticles increases their sizes as compared to the initial nanoparticle dimensions measured in the absence of catalase. The largest particle sizes (between 484 nm and 530 nm) were reached upon augmentation of the fish oil and curcumin contents. Moreover, a presence of two populations of nanocarriers is established in the dispersed systems (Table 2). The two populations represent a coexistence of large-size (∼500 nm) particles and smaller-size (∼160 nm) particles. For cubosomal formulations of lipids, it has been suggested that they correspond to coexisting cubosomes and small vesicles or precursors of intermediate-type liquid crystalline structures [34,39]. The inclusion of CAT protein appears to favor the cubosome nanoparticle population.

### 2.2. In Vitro Evaluation of Catalase- and Curcumin-Loaded Liquid Crystalline Nanocarriers

#### 2.2.1. Viability of Cubosome Nanoparticle-Treated Differentiated SH-SY5Y Cells

**Figure 6:**
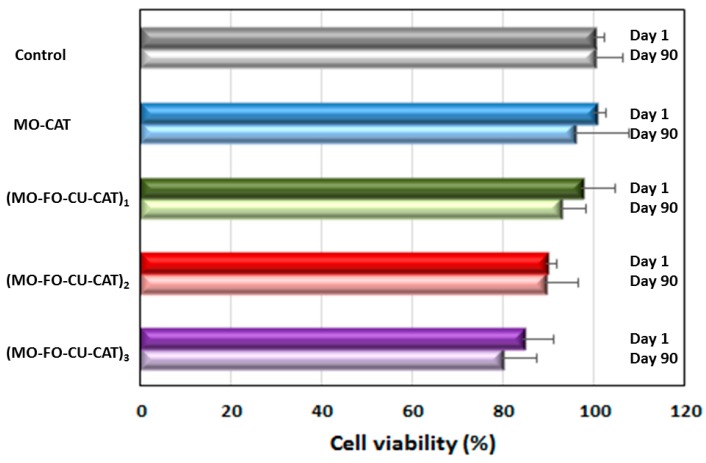
Cellular viability of retinoic acid-differentiated SH-SY5Y cells after 24 h exposure to 0.5 µM of catalase-loaded liquid crystalline nanoparticles. The histograms at Day 1 and Day 90 correspond to the time elapsed (one day or 90 days) after the nanoparticles’ preparation before cell exposure. The MO-FO-CU-CAT compositions are presented in Section 4.3 of Methods.

The human neuroblastoma SH-SY5Y cells were differentiated by 10 μM of retinoic acid (RA) for 5 days in order to obtain extensive proliferation of neurites and reduced cell body sizes, which are typical for a neuronal cell phenotype [19,71,72]. Then, the cells were exposed to 0.5 µM of catalase-loaded cubosome nanocarriers during 24 h. Freshly prepared nanoparticles (one day after NP preparation) and 90 days-stored NPs were investigated for their impact on the cellular viability. Unexposed cells were used as viability controls. The obtained 3-(4,5-Dimethylthiazol-2-yl)-2,5-diphenyl tetrazolium bromide (MTT) data (Figure 6) demonstrate that the cellular viability decreases from 100 ± 2.2% to 84.5 ± 6.7% for catalase-loaded cubosome particles, which contain increasing amounts of incorporated fish oil and curcumin (see Section 4.3 of Methods). This decrease in cellular viability was not significant (*p* < 0.05) compared to the control. The MTT test indicated the safety of the dual drug-loaded nanoparticles. In addition, the data obtained with the 90-days-stored nanoparticles did not show a significant viability difference as compared to the newly prepared nanoparticles. This result evidenced the stability of the studied cubosome nanoparticles with 90 days of storage.

#### 2.2.2. Catalase Peroxidatic Activity in Cell Lysates of Differentiated SH-SY5Y Cells Obtained after Treatment with Cubosome Nanoparticles

**Figure 7:**
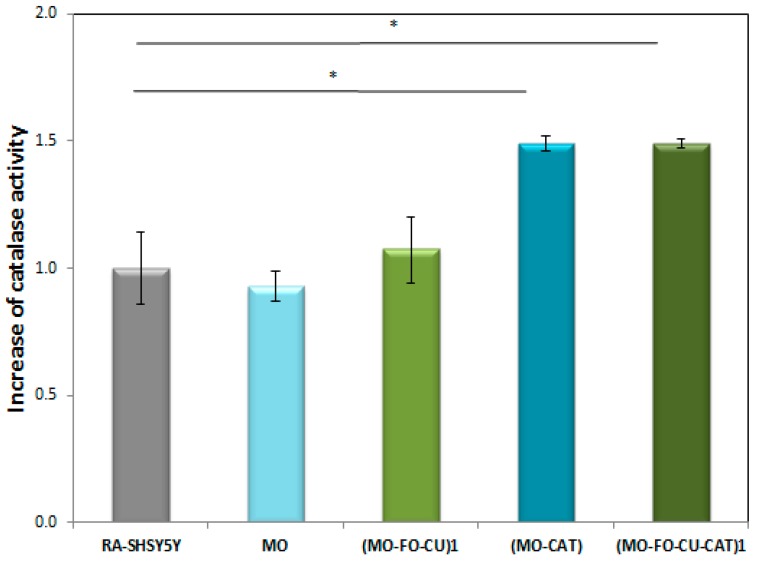
Fold increase in catalase enzymatic activity (peroxidatic function) determined in supernatants of cell lysates of differentiated SH-SY5Y cells (RA-SH-SY5Y) after exposure to blank nanoparticles (MO), catalase-loaded (MO-CAT) cubosome nanoparticles, dual drug-loaded (MO-FO-CU) or enzyme-loaded (MO-FO-CU-CAT) cubosome nanoparticles.

The successful delivery and uptake of blank and catalase-loaded cubosome nanoparticles in RA-differentiated SH-SY5Y cells was evaluated by determining catalase peroxidatic activity [48,49] in supernatants of cell lysates generated after cubosomal treatment. Catalase is a ubiquitous antioxidant enzyme involved in the detoxification of H_2_O_2_ (a toxic product of the normal aerobic metabolism or of pathogenic ROS production). One unit of enzymatic reaction activity is defined as the amount of enzyme that will cause the formation of 1 nmol of formaldehyde per minute at 25 °C. It serves for the quantification of the cytosolic catalase. The determined catalase peroxidatic activity in lysates of non-treated RA-SH-SY5Y cells, used as a control, was 15.37 ± 0.14 nmol/min/mL. Figure 7 shows that there was no significant difference in the catalase activity between the control and the cells exposed to blank nanoparticles (MO). The measured value of 14.47 ± 0.06 nmol/min/ml was close. A tendency for the catalase activity to increase was observed for RA-SH-SY5Y cells exposed to FO- and CU-loaded nanoparticles (MO-FO-CU)_1_. The peroxidatic activity value raised to 16.51 ± 0.13 nmol/min/mL. The results with cells exposed to catalase-loaded nanoparticles (MO-CAT) demonstrated a significant increase in the catalase peroxidatic activity (activity value equal to 27.09 ± 0.02 nmol/min/mL, *p* < 0.05). An increase in the peroxidatic activity was observed also with catalase- and curcumin-loaded nanoparticles (MO-FO-CU-CAT)_1_ (Figure 7). This implies that catalase is delivered by the liquid crystalline nanocarriers inside the neuronally derived cells. Noticeably, the cellular uptake of CAT-loaded nanoparticles results in an overall increase in the measured enzymatic activity (peroxidatic function).

## 3. Discussion

### 3.1. Structural Effect of Catalase Entrapped in Curcumin-Loaded Self-Assembled Liquid Crystalline Nanocarriers

We determined by BioSAXS the different kinds of mesophase structures obtained in MO/TPEG_1000_/FO/CU/CAT self-assembled systems of therapeutic interest (Figure 3, Figure 4 and Figure 5). Whereas diamond *Pn3m* cubic structures were present in the MO/TPEG_1000_ assemblies, the cubic liquid crystalline architectures identified in the MO/TPEG_1000_/FO/CU/CAT systems were of either *Pn3m* or *Im3m* space group symmetries. Thus, the inclusion of catalase did not disrupt the overall cubic liquid crystalline organization of the curcumin-loaded nanocarriers. However, intermediates of the cubic-to-sponge mesophase transition were observed with increasing fish oil content at a temperature of 22 °C.

At a low co-lipid content (fish oil/monoolein ratio 10:90 wt%/wt%), the bulk MO/TPEG_1000_/FO/CU/CAT systems displayed *Pn3m* and *Im3m* cubic phases (Figure 4a). Intermediates of the cubic-to-sponge mesophase transition appeared at FO/MO ratios of 20:80 and 30:70 (wt%/wt%) (Figure 4b,c). A predominant sponge phase was obtained at a high content of fish oil (FO/MO ratio 40:60 wt%/wt%) (Figure 4d). However, the cubic structure was retained at 90%wt water content despite the elevated percentage of fish oil at DL 40:60 (Figure 4d). Therefore, the hydration level was crucial for the resulting mesoscale organization of the mixed assemblies.

The observed coexistence of two cubic domains in the nanocarriers with intermediate fish oil content (e.g., FO/MO ratio 20:80, Table 1) may be due to the insufficient amount of co-lipid necessary to trigger a phase transition to a new mesophase type. Thus, the overall *Pn3m* cubic structure will contain coexisting domains, which are more rich or less rich in a co-lipid ingredient or in a PEGylated amphiphile. In a previous study, we showed that the inhomogeneous distribution of the PEGylated surfactant along the monoolein membrane can result in the coexistence of cubic phase nanodomains with different lattice parameters, but the same space group (*Pn3m*) symmetry [39]. The domains with the smaller cubic unit cell dimension corresponded to the hydrated pure monoolein lipid, and the larger cubic unit cell corresponded to the mixed lipid assembly. Similar effects of inhomogeneous distribution of lipid components or nanodomain formation are observed in some of the samples studied in the present work. This yields two different magnitudes of the cubic lattice parameters, which characterize the coexisting *Pn3m* cubic phase domains (e.g., a_1_(_Q_) = 16.4 nm and a_2_(_Q_) = 20.0 nm).

Nano-dispersions of liquid crystalline particles were obtained with the MO/TPEG_1000_/FO/CU/CAT mixtures in the excess water (95 wt%) phase. Their internal organization involved *Pn3m* and *Im3m* cubic structures in the absence of catalase (Figure 5) and the induction of primitive *Im3m* cubic structures in the catalase-loaded nanoparticles. The cubic unit cell dimensions increased in the presence of an associated catalase enzyme, while retaining the overall cubic phase organization. The structural influence of protein incorporation into liquid crystalline lipid assemblies has been discussed in several publications [8,9,11,20,21,22,23,24,25,26,27,28]. For instance, neurotrophin BDNF confinement resulted in the formation of multiphase and multicompartment liquid crystalline lipid nanoparticles [11]. The transmembrane β-barrel BamA protein caused an increase in the lattice parameter of the host lipid cubic phase upon encapsulation [26]. By contrast, the lipo-protein BamB–E caused the cubic lattice parameter to decrease [26]. The effect of amphiphilic and soluble proteins on the nanochannel diameters in bicontinuous cubic *Pn3m* phases of monoolein has received considerable attention as well [8,11,23,25]. Long-living swollen states, corresponding to a diamond cubic phase with large water channels, have been stabilized in some cases [2,30]. Cryo-TEM and freeze-fracture electronic microscopy imaging has indicated the inclusion of proteins into nanopockets of the supramolecular cubosomic assemblies and the induction of nanodomains [8,11,25]. In the present study, catalase was incorporated in the cubosome carriers under excess water conditions. The lattice parameters of the curcumin-loaded cubosomes were between 16.4 nm (*Pn3m* space group) and 21.3 nm (*Im3m* space group), depending on the amount of fish oil and curcumin in the mixtures. The a_(Q)_ values increased to 23.2–26.3 nm (*Im3m* space group) upon the addition of catalase. This implies that the hydrated enzyme catalase causes swelling of the cubosomal network architectures, rather than their dehydration.

### 3.2. Catalase Peroxidatic Function Following Cellular Treatment with Dual Drug-Loaded Cubosomes

**Figure 8:**
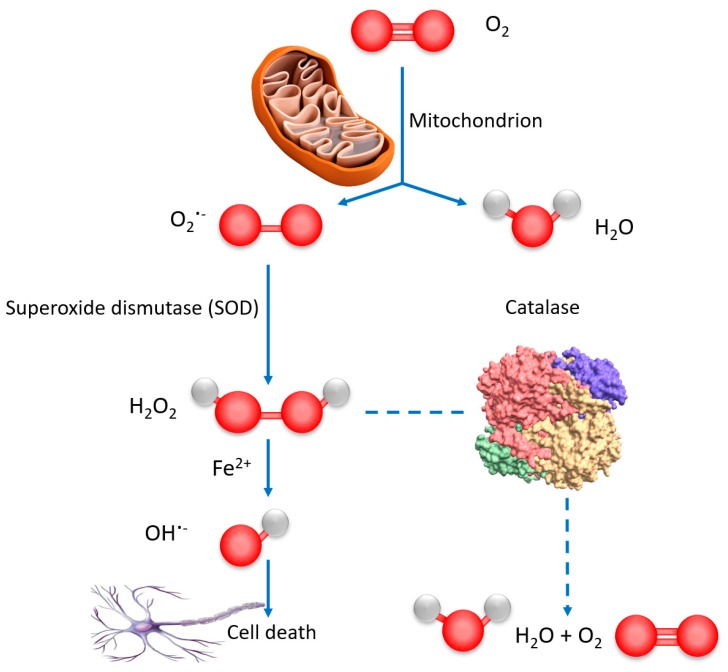
Scheme of reactive oxygen species (ROS) generation and organism self-defense by natural enzymes. The generated ROS levels are principally linked to mitochondria. The superoxide radicals O_2_^−^ are converted to less toxic H_2_O_2_ by the enzyme superoxide dismutase (SOD). In the presence of Fe^2+^, some of the H_2_O_2_ molecules can be reduced to highly reactive OH**^−^** ROS, which attacks various biomolecules (proteins, DNA, and lipids) and causes cell death. Catalase blocks that pathway and saves the organisms by decomposing H_2_O_2_ into harmless water and oxygen [44].

Catalase plays a crucial role in the adaptive response to hydrogen peroxide as ROS [44,55]. Human catalase belongs to the family of catalases, which catalyze the dismutation of H_2_O_2_ into water and molecular oxygen (Figure 8). In addition to its dominant catalytic function (decomposition of H_2_O_2_), catalase can also decompose peroxynitrite and oxidize nitric oxide to nitrogen dioxide. It exhibits marginal peroxidase activity (i.e., oxidation of organic substrates with concomitant reduction of peroxide) and low oxidase activity (O_2_-dependent oxidation of organic substrates) [44,47]. At a high concentration of H_2_O_2,_ the catalytic pathway starts. At a low concentration of H_2_O_2,_ the peroxidatic pathway is initiated [48,49], in which various hydrogen donors such as alcohols, phenols, hormones, heavy metals and nitrite (serving as the second molecule that assures the role of H_2_O_2_) are oxidized [44].

Low levels of catalase expression correlate with a high production of H_2_O_2_ [53,54]. As a consequence, this effect causes the activation of signaling pathways associated with different diseases including Alzheimer‘s disease [51,52,53,54]. Our work provides experimental evidence that catalase-loaded cubosome nanoparticles are promising candidates for the intracellular delivery of the unstable protein towards treatment or prevention of neurodegenerative disorders. Catalase, like many enzymes, is unstable in aqueous phase and shows a propensity to aggregate. This may lead to a loss of activity [61]. In our strategy, the catalase-loaded cubosome nanoparticles protect the enzyme and ensure its intracellular uptake.

**Figure 9:**
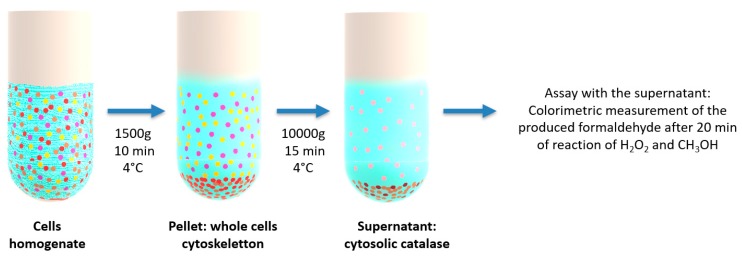
Schematic protocol employed in the catalase enzymatic activity assay. The assay is performed with supernatants of cell lysates, which contain the cytosolic catalase. The cell lysates are first subjected to differential centrifugation, and the colorimetric measurements of the CAT peroxidatic activity are taken with the resulting supernatants.

We analyzed the peroxidatic function of catalase for the quantification of its activity in lysates from RA-differentiated SH-SY5Y cells exposed to blank nanoparticles (NPs), curcumin (CU)-loaded NPs, catalase (CAT)-loaded NPs, and dual (CAT-CU)-loaded NPs. The enzymatic activity was determined by colorimetrical measurements of formaldehyde (Figure 9) formed thanks to the peroxidatic function of catalase [48,49]. The results shown in Figure 7 indicate an increased catalase activity in supernatants of cells exposed to catalase-loaded nanoparticles (i) without curcumin (MO-CAT) or (ii) with dual loading of CAT and curcumin (MO-FO-CU-CAT). The enhanced enzymatic activity can be explained by an increase in the amount of cytosolic catalase in the cells treated with CAT-loaded nanoparticles. This confirms that the cubosome nanoparticles provide an efficient delivery and uptake of the catalase enzyme into the cells.

We established that the curcumin-containing dual-drug loaded nanoparticles (MO-FO-CU-CAT) maintain or increase the CAT activity (Figure 7), unlike other antioxidants, which can inhibit the catalase enzymatic function [56,57]. Molecular dynamic simulations have demonstrated that curcumin can significantly increase the activity of bovine liver catalase (BLC) as it favors the access of the substrate to the active site of the enzyme [59]. The enzymatic activity has been suggested to increase through a curcumin-triggered re-arrangement of the amino acid residues in the structural pocket of catalase. The increased distances between the residues of the formed channel enable a larger amount of substrate to reach the active site. The entrance space increased from 250 Å to 440 Å, which essentially facilitated the substrate’s access to the enzyme active pocket. Curcumin may also increase the amount of α-helical content in BLC, leading to the stabilization of the protein’s secondary structure [59,60].

In conclusion, dual drug-loaded nanocarriers of the cubosome type were obtained and were characterized by stable mesophase organization during three months of storage. The catalase- and curcumin loaded (MO-FO-CU-CAT) cubosome nanoparticles efficiently delivered the therapeutic molecules inside the neuronally derived SH-SY5Y cells as evidenced by the increased activity of the antioxidant enzyme. The cubosomal nanoarchitectures preserved the encapsulated enzyme (CAT) in a functional state, ensuring the cell’s defense against reactive oxygen species (catalytic and peroxidatic functions). Moreover, the dual-loaded cubosomes provided an enhanced activity of catalase in differentiated SH-SY5Y cells. Further studies are needed in order to determine an eventual synergistic antioxidant effect of catalase and curcumin upon dual delivery by liquid crystalline nanocarriers.

## 4. Materials and Methods

### 4.1. Materials

Curcumin (CU), fish oil (FO), monoolein (MO), and d-α-tocopherol polyethylene glycol-1000 succinate (a pegylated amphiphile denoted as TPEG_1000_) were purchased from Sigma-Aldrich (Lyon, France). For cell culture experiments, Dulbecco’s modified Eagle’s Medium (DMEM), streptomycin-penicillin, phosphate buffered saline (PBS), trypsin, ethylenediaminetetraacetic acid EDTA, retinoic acid (RA) and 3-(4,5-Dimethylthiazol-2-yl)-2,5-diphenyl tetrazolium bromide (MTT) were supplied by Sigma-Aldrich. Foetal bovine serum (FBS) was provided by Thermo Fischer Scientific (Illkirch, France). The water used was of MilliQ quality (Millipore Corp., Molsheim, France).

### 4.2. Preparation of Bulk Liquid Crystalline MO/TPEG_1000_/Fish Oil/Curcumin Systems

The lipid monoolein (MO), the surfactant TPEG_1000_, fish oil (FO), and curcumin (CU) were weighed and dissolved in chloroform. The samples were prepared at room temperature at four different fish oil/monoolein weight ratios of 10:90, 20:80, 30:70 and 40:60 (wt%/wt%) (Table 3). The solvent was evaporated under a stream of a nitrogen gas for 1 h at room temperature to create a thin film lipid sample. The samples were lyophilized overnight under cooling to remove the excess solvent. This step was followed by the hydration of the thin film samples by a solution of catalase (0.5 wt%) protein (a buffer solution with pH 7 prepared using Milli-Q water). The concentrations were varied from 50 wt% to 90 wt% in aqueous phase with regard to the lipid phase. Finally, the samples were vortexed vigorously at room temperature in cycles during 15 min.

### 4.3. Preparation of Aqueous Dispersions of Nanoparticles

The lipid nanoparticles were prepared by the method of hydration of a lyophilized thin lipid film followed by physical agitation in excess aqueous phase [19,22,34,39]. The lipids and the hydrophobic constituents were dissolved in chloroform and mixed at desired proportions (Table 4). The solvent in the vials was evaporated under a stream of nitrogen gas for 1 h at room temperature to create a thin film lipid sample. The excess organic solvent was removed overnight using a lyophilizer. The thin film samples were hydrated for 24 h at room temperature in a buffer solution for blank (catalase-free) lipid nanoparticles and in a solution of catalase (0.5 wt%) for CAT-loaded lipid nanoparticles. The self-assembled mixtures were dispersed using a vortex until milky solutions were obtained.

### 4.4. Synchrotron Small Angle X-Ray Scattering (BioSAXS)

For nanostructure determination with lipid/protein assemblies, BioSAXS experiments were performed at the SWING beamline [70] of synchrotron SOLEIL (Saint Aubin, France). The sample-to-detector distance was 3 m. The patterns were recorded with a two-dimensional EigerX 4-M detector (Dectris, Baden, Switzerland) at 12 keV, allowing measurements in the *q*-range from 0.00426 to 0.37 Å^−1^. The *q*-vector was defined as *q* = (4π/λ) sin θ, where 2θ is the scattering angle. The synchrotron radiation wavelength was λ = 1.033 Å. The *q*-range calibration was done using a standard sample of silver behenate (*d* = 58.38 Å). The temperature was 22 °C.

The investigated samples were filled in capillaries with a diameter of 1.5 mm and were sealed by paraffin wax. They were oriented in front of the X-ray beam (25 × 375 μm^2^) using a designed holder for multiple capillaries positioning (X, Y, Z). Exposure times of 500 ms (for bulk lipid samples) or 1 s (for diluted nanoparticles) were used. No radiation damage was observed at these exposure times. Scattering patterns of an empty capillary and a capillary filled with MilliQ water were recorded for intensity background subtraction. Data processing of the recorded 2D images was performed by the FOXTROT software [73]. An average of three spectra per capillary was acquired.

The lattice parameters of the liquid crystalline phases were derived from the Bragg peaks detected in the X-ray diffraction patterns. The assigned reflections were fitted through the Miller indexes according to the following relationships:

*a/d* = 1,2,3, …  for structures with a lamellar spacing (2)

(*a/d*)^2^ = 2,3, 4, 6, 8, 9, 10, 11, 12, 14, … for the *Pn3m* space group (Diamond cubic, D) (3)

(*a/d*)^2^ = 2,4, 6, 8, 10, 12, 14, 16, 18, … for the *Im3m* space group (Primitive cubic, P) (4)

(*a/d*)^2^ = 6, 8, 14, 16, 20, 22, 24, 26, … for the *Ia3d* space group (Gyroid cubic, G) (5)

### 4.5. Nanoparticle Size Determination

The hydrodynamic diameters of the particles in the nanodispersions were determined based on the principle of quasi-elastic light scattering (QELS). The particle size distribution was determined by means of a Nano-ZS90 device (Malvern Instruments) collecting the intensity of the scattered light at an angle of 90° with regards to the incident laser beam. Data collection was carried out at 25 °C. The samples were diluted to 1/10 in a buffer in order to ensure Brownian motion conditions for the particles. The refractive index and viscosity of the MilliQ water wree equal to 1.330 and 0.8872, respectively. Each analysis was a result of three consecutive measurements.

### 4.6. Cell Culture

The human neuroblastoma SH-SY5Y cells were cultured in DMEM medium with high glucose supplement, 10% FBS, and 0.5% streptomycin-penicillin. They were incubated at 37 °C in a saturated humidity atmosphere containing 5% CO_2_. Before every experiment, the cells were grown in plastic Nunc cell culture flasks (75 cm^2^) (Thermo Scientific, Illkirch, France) and were treated with 10 μM retinoic acid (RA) for 5 days towards differentiation into neuronal cells [19,40,71,72]. The adherent SH-SY5Y cells were divided twice weekly with the use of 0.05% trypsin-EDTA for up to 5 min, followed by centrifugation (200× *g*) at 4 °C for 5 min. The cells were counted using a KOVA® cell counting chamber (VWR, Fontenay-sous-Bois, France), and seeded at densities of 2 × 10^4^ cells/well in 96-well plates or 10^6^ cells/flask (25 cm^2^) (depending on the type of biological analysis to be carried out). After 24 h, the SH-SY5Y cells were incubated with RA (10 μM) for 5 days, changing the medium with RA at least once. The neuronal phenotype was distinguished by the extensive proliferation of neurites [71,72].

### 4.7. Cell Viability

The cell viability was determined by the tetrazolium salt test (3-(4,5-dimethylthiazol-2-yl) -2,5-diphenyl tetrazolium bromide, MTT) [19,40]. The solution of MTT was prepared in PBS and was filtered prior to use. This reagent is reduced to formazan by the mitochondrial succinate dehydrogenase enzyme in living cells. The MTT compound forms a purple precipitate, the quantity of which is proportional to the metabolic activity of the living cells. The cells were seeded at a density of 20 × 10^4^ cells/well in 96-well plates. After 5 days of treatment with 10 μM retinoic acid, cubosome nanoparticles were incubated with the cells at lipid concentrations of 0.5 µM at 37 °C for 24 h. Untreated cells maintained in DMEM medium were used as controls. MTT was added at a concentration of 5 mg/mL at 37 °C. After 1 h of incubation of the cells with MTT, the medium was removed, and the cells were dissolved in 100% DMSO to solubilize the formazan precipitate. The optical density was then measured at 570 nm by a microplate reader. The quantification was done using measurements of a minimum of six wells.

### 4.8. Catalase Enzymatic Activity (Peroxidatic Function) in Supernatants of Cell Lysates

Catalase (CAT) activity was measured in SH-SY5Y cell lysates in order to evaluate the effect of the liquid crystalline cubosomal nanoparticle treatment on the enzymatic function. CAT catalyzes the dismutation of two molecules of hydrogen peroxide into molecular oxygen and two molecules of water, according to relationship (6). This enzyme acts at higher concentrations of hydrogen peroxide than the enzyme peroxidase. CAT also exhibits peroxidatic activity, presented by relationships (7) and (8) below.

Here, SH-SY5Y cells were seeded at a density of 10^6^ cells in 25 cm^2^ culture flasks containing 5 ml of DMEM medium. After 24 h, the cell culture medium was replaced by 10 µM of RA solution for 5 days of incubation. Then, aqueous dispersions of fish oil, curcumin, catalase or lipid nanoparticles (0.5 µM) were introduced in the FBS-free medium. After 24 h incubation, the cells were collected by centrifugation at 1500× *g* for 10 min at 4 °C. The cell pellet was homogenized on ice in 1 ml of cold buffer of potassium phosphate. Then, the samples were centrifuged at 10000× *g* for 15 min at 4 °C. The assay was performed with the supernatants according to the instructions of the Cayman’s Catalase Assay Kit (catalogue No. 707002) (Cayman Chemical, Ann Arbor, MI, USA), which utilizes the peroxidatic function of CAT for determination of the enzyme activity.

CAT catalytic activity:  2 H_2_O_2_  **-------catalase------>**  2 H_2_O + O_2_ (6)

CAT peroxidatic activity:  H_2_O_2_ + AH_2_  **------catalase------>**  2 H_2_O + A (7)

where AH_2_ and A represent the substrate, i.e., low molecular weight aliphatic alcohols serving as electron donors. In the present methodology,

CAT peroxidatic activity: H_2_O_2_ + CH_3_OH **-----catalase------>** 2 H_2_O + CH_2_O (8)

This method is based on the reaction of the enzyme CAT with methanol in the presence of an optimal concentration of H_2_O_2_ according to equation (8). The produced formaldehyde was measured colorimetrically with Purpald as the chromogen. The absorbance was monitored at 540 nm using a plate reader.

### 4.9. Statistical Analyses

The data are presented as the mean values of standard deviation (SD) of three independent experiments. The results were analyzed by the Tukey test after one-way analysis of variance (ANOVA). The probability values *p* < 0.05 were considered statistically significant across the treatment groups.

## Figures and Tables

**Table 1:** Internal liquid crystalline structure types and lattice parameters of MO/TPEG_1000_/FO/CU/CAT self-assembled architectures determined from BioSAXS analysis of the data shown in Figure 4.

Samples	Liquid Crystalline Structures	Lattice a(_Q_) (nm)
C196	*Pn3m* cubic	18.5
C197	*Pn3m* cubic	18.9
C199	*Im3m* cubic	21.7
C286	*Pn3m* cubic	20.0
C287	Cubic intermediate	-
C288	Coexisting *Pn3m* cubic domains	20.0/22.7 ^a^
C376	Cubic intermediate	-
C377	Cubic intermediate	-
C378	*Pn3m* cubic	20.7
C379	*Im3m* cubic	22.2
C466	Cubic intermediate	-
C467	Cubic intermediate	-
C468	Cubic intermediate—sponge	-
C469	*Pn3m* cubic	17.5

^a^ Coexistence of two cubic structures.

**Table 2:** Unit cell lattice parameters and size distributions of catalase-free and catalase-loaded MO/FO/CU cubosome nanoparticles (NPs) stabilized by TPEG_1000_. The cubic space group type is indicated in Figure 5 for every sample.

Nanoparticles	Lattice a(_Q_) (nm) ^a^	NPs’ Size (nm) ^b^
**Curcumin-Loaded NPs**		
MO-TPEG_1000_	1	106
(MO-FO-CU)_1_	16.4/20.0 ^c^	106/220 ^d^
(MO-FO-CU)_2_	20.0	220
(MO-FO-CU)_3_	21.3	255
**Curcumin and Catalase-Loaded NPs**		
MO-CAT	-	484
(MO-FO-CU-CAT)_1_	23.2	164/550 ^b^
(MO-FO-CU-CAT)_2_	25.0/26.3 ^a^	150/459 ^b^
(MO-FO-CU-CAT) _3_	26.3	164/531 ^b^

^a^ Determined by BioSAXS; ^b^ determined by QELS; ^c^ coexistence of two structures; ^d^ coexistence of two populations of NPs.

**Table 3:** Compositions of bulk liquid crystalline phases presented as mass proportions of MO, TPEG_1000_, FO, CU and catalase.

Sample Code	Catalase (0.5 wt%) in Aqueous Buffer (pH 7) (g)	MO (g)	TPEG_1000_ (g)	FO (g)	CU (g)
	**Dilution Line (FO:MO) = DL (10:90)**				
**C195**	0.013	0.0090	0.0020	0.0010	0.00013
**C196**	0.015	0.0072	0.0018	0.0009	0.00010
**C197**	0.018	0.0054	0.0013	0.0007	0.00008
**C199**	0.023	0.0018	0.0004	0.0002	0.00003
	**Dilution Line (FO:MO) = DL (20:80)**				
**C285**	0.013	0.0080	0.0020	0.0022	0.00025
**C286**	0.015	0.0066	0.0016	0.0016	0.00018
**C287**	0.018	0.0050	0.0012	0.0011	0.00013
**C288**	0.020	0.0032	0.0008	0.0009	0.00010
	**Dilution Line (FO:MO) = DL (30:70)**				
**C376**	0.015	0.0056	0.0014	0.0027	0.00030
**C377**	0.018	0.0042	0.0010	0.0020	0.00023
**C378**	0.020	0.0028	0.0007	0.0013	0.00015
**C379**	0.023	0.0014	0.0003	0.0007	0.00008
	**Dilution Line (FO:MO) = DL (40:60)**				
**C466**	0.015	0.0048	0.0012	0.0036	0.00040
**C467**	0.018	0.0036	0.0009	0.0027	0.00030
**C468**	0.020	0.0025	0.0006	0.0017	0.00019
**C469**	0.023	0.0012	0.0003	0.0009	0.00010

**Table 4:** Lipid nanoparticle constituents and their mass proportions.

95 wt% Aqueous Phases		MO (g)	TPEG_1000_ (g)	FO (g)	CU (g)
CU-Loaded NPs	CAT-Loaded NPs				
MO	MO-CAT	0.02	0.005		
MO_1_-FO_1_		0.016	0.004	0.0045	
(MO-FO-CU)_1_	(MO-FO-CU-CAT)_1_	0.016	0.004	0.0045	0.0005
(MO-FO-CU)_2_	(MO-FO-CU-CAT)_2_	0.012	0.003	0.0090	0.0010
(MO-FO-CU)_3_	(MO-FO-CU-CAT)_3_	0.008	0.002	0.0135	0.0015
